# Exosomes from Human Placenta Choriodecidual Membrane-Derived Mesenchymal Stem Cells Mitigate Endoplasmic Reticulum Stress, Inflammation, and Lung Injury in Lipopolysaccharide-Treated Obese Mice

**DOI:** 10.3390/antiox11040615

**Published:** 2022-03-23

**Authors:** Milton D. Chiang, Chao-Yuan Chang, Hung-Jen Shih, Van Long Le, Yen-Hua Huang, Chun-Jen Huang

**Affiliations:** 1 International Ph.D. Program in Medicine, College of Medicine, Taipei Medical University, Taipei 110, Taiwan; d142108016@tmu.edu.tw (M.D.C.); drlong.gmhshue@gmail.com (V.L.L.); 2 Department of Medical Research, Wan Fang Hospital, Taipei Medical University, Taipei 116, Taiwan; yuanc669@gmail.com; 3 Integrative Research Center for Critical Care, Wan Fang Hospital, Taipei Medical University, Taipei 116, Taiwan; 4 Graduate Institute of Clinical Medicine, College of Medicine, Taipei Medical University, Taipei 110, Taiwan; 5 Division of Urology, Department of Surgery, Changhua Christian Hospital, Changhua 500, Taiwan; jasta1206@gmail.com; 6 Department of Recreation and Holistic Wellness, MinDao University, Changhua 523, Taiwan; 7 Department of Urology, School of Medicine, College of Medicine, Taipei Medical University, Taipei 110, Taiwan; 8 Department of Anesthesiology and Critical Care, Hue University of Medicine and Pharmacy, Hue City 52000, Vietnam; 9 Department of Biochemistry and Molecular Cell Biology, School of Medicine, College of Medicine, Taipei Medical University, Taipei 110, Taiwan; rita1204@tmu.edu.tw; 10 Research Center for Cell Therapy and Regeneration Medicine, Taipei Medical University, Taipei 110, Taiwan; 11 International Ph.D. Program for Cell Therapy and Regeneration Medicine, College of Medicine, Taipei Medical University, Taipei 110, Taiwan; 12 Center for Reproductive Medicine, Taipei Medical University Hospital, Taipei Medical University, Taipei 110, Taiwan; 13 Department of Anesthesiology, Wan Fang Hospital, Taipei Medical University, Taipei 116, Taiwan; 14 Department of Anesthesiology, School of Medicine, College of Medicine, Taipei Medical University, Taipei 110, Taiwan

**Keywords:** lung injury, obesity, endoplasmic reticulum stress, exosomes, LPS

## Abstract

Endoplasmic reticulum (ER) stress mediates the effects of obesity on aggravating sepsis-induced lung injury. We investigated whether exosomes from human placenta choriodecidual membrane-derived mesenchymal stem cells (pcMSCs) can mitigate pulmonary ER stress, lung injury, and the mechanisms of inflammation, oxidation, and apoptosis in lipopolysaccharide-treated obese mice. Diet-induced obese (DIO) mice (adult male C57BL/6J mice fed with a 12-week high-fat diet) received lipopolysaccharide (10 mg/kg, i.p.; DIOLPS group) or lipopolysaccharide plus exosomes (1 × 10^8^ particles/mouse, i.p.; DIOLPSExo group). Our data demonstrated lower levels of ER stress (upregulation of glucose-regulated protein 78, phosphorylated eukaryotic initiation factor 2α, and C/EBP homologous protein; *p* = 0.038, <0.001, and <0.001, respectively), inflammation (activation of nuclear factor-kB, hypoxia-inducible factor-1α, macrophages, and NLR family pyrin domain containing 3; upregulation of tumor necrosis factor-α, interleukin-1β, and interleukin-6; *p* = 0.03, <0.001, <0.001, <0.001, <0.001, <0.001, and <0.001, respectively), lipid peroxidation (*p* < 0.001), and apoptosis (DNA fragmentation, *p* = 0.003) in lung tissues, as well as lower lung injury level (decreases in tidal volume, peak inspiratory flow, and end expiratory volume; increases in resistance, injury score, and tissue water content; *p* < 0.001, <0.001, <0.001, <0.001, <0.001, and =0.002, respectively) in the DIOLPSExo group than in the DIOLPS group. In conclusion, exosomes from human pcMSCs mitigate pulmonary ER stress, inflammation, oxidation, apoptosis, and lung injury in lipopolysaccharide-treated obese mice.

## 1. Introduction

Acute lung injury induced by sepsis is an inflammatory pulmonary disease with a complex mechanism, and it is a life-threatening illness that creates major health-care challenges worldwide [1]. Notably, effective therapy against sepsis-induced acute lung injury remans lacking to date [1]. In recent years, concerns over the effects of obesity on the incidence and outcomes of sepsis-induced acute lung injury are increasing because of the widespread prevalence of obesity [2]. Obesity has adverse impacts on lung function, including decreased lung volumes and expiratory flow rates that are more susceptible to bacterial invasion [3]. Moreover, due to an insufficient blood supply to the growing adipose tissues, obesity is associated with oxidative stress [4]. As oxidative stress activates the upstream transcription factors nuclear factor-κB (NF-κB) and hypoxia-inducible factor-1α (HIF-1α), enhanced inflammation and macrophage activation are observed in obesity [4]. In addition, expanding adipose tissues also cause adipocyte death (e.g., apoptosis) and mechanical stress that can further trigger inflammation [4]. As such, obesity has an adverse impact on acute lung injury development and may worsen the outcomes of acute lung injury induced by sepsis [5,6]. Similar to non-obesity, currently there is no effective sepsis-induced acute lung injury therapy approach in obesity [6].

The endoplasmic reticulum (ER) is the primary location of protein synthesis, maturation, and secretion [7]. When encountering an imbalanced redox state, disruption of calcium homeostasis, viral infections, and/or bacterial infections, ER may lose its homeostasis and result in ER stress [5,7,8,9]. A signal response known as the unfolded protein response (UPR) will then be activated to restore ER function [5,7,8,9]. However, UPR is a double-edged sword. If ER stress is beyond its compensatory capacity (e.g., with sepsis), UPR can switch from cyto-protection to become a threat and lead to cellular dysfunction and cell death, e.g., apoptosis [10,11]. It is well established that the ER chaperone glucose-regulated protein 78 (GRP78), eukaryotic translation-initiation factor 2α (eIF2α), and C/EBP homologous protein (CHOP) are crucial for mediating the detrimental effects of ER stress [12,13,14,15]. Notably, ER stress plays a crucial role in mediating sepsis-induced acute lung injury [16,17]. In addition to sepsis, obesity activates ER stress [18,19]. Moreover, study data indicated that ER stress is involved in mediating the effects of obesity on aggravating sepsis-induced organ injury [3,5]. Inhibition of ER stress thus may confer certain protective effects and mitigate sepsis-induced acute lung injury in obesity [5].

Mesenchymal stem cells (MSCs) are stem cells that can be amplified and differentiated without limitation [20]. Moreover, MSCs possess potent therapeutic effects against sepsis, as preclinical data indicated that MSCs can improve survival and mitigate acute lung injury induced by sepsis [21]. Notably, the therapeutic effects of MSCs depend mainly on intercellular communication that mediated by exosomes, namely the nano-sized lipid bilayer-enclosed extracellular vesicles secreted by MSCs [22,23,24]. Exosomes contain functional cargos from MSCs, including proteins, peptides, lipids, cytokines, messenger RNAs (mRNAs), noncoding RNAs (e.g., microRNAs, miRNAs), etc. [23,24]. Through transferring the functional cargos from MSCs to the recipient cells (i.e., intercellular communication), exosomes are responsible for the therapeutic effect of MSCs [23,24]. Exosomes have cup forms of 30–150 nm diameter, as seen by transmission electron microscopy (TEM), and may be sterilized by filtering [23,24]. Exosomes are smaller and less complex than their parent cells, making them easier to generate and store. They are also important non-toxic transporters with low immunogenicity [23,24]. Therefore, exosomes can be an essential alternative due to their advantages over their parent MSCs.

The therapeutic potentials of exosomes from MSCs (e.g., adipose-derived and bone marrow-derived MSCs) against sepsis have also been demonstrated. For instance, exosomes from MSCs can improve survival and mitigate sepsis-induced acute lung injury in non-obese mice [25,26]. Similar to non-obese mice, our recent data demonstrated the therapeutic effects of exosomes from MSCs on mitigating sepsis-induced liver injury in obese mice [27]. However, the effects of exosomes from MSCs on modulating sepsis-induced acute lung injury in obese mice remains unstudied. To elucidate further on this issue, we conducted this murine study with the hypothesis that exosomes from MSCs can mitigate sepsis-induced acute lung injury in obese mice.

It is established that bacterial Gram-negative infection is one of the primary causes of sepsis-induced acute lung injury, and lipopolysaccharide in the external membranes of Gram-negative bacteria can induce lung damage and inflammatory responses [28]. Experiments in animals revealed that lipopolysaccharide administration significantly increases inflammatory cell infiltration, oxidative stress, and cell death (e.g., apoptosis) in lung tissues [29]. This study thus chose to employ lipopolysaccharide to induce acute lung injury in obese mice. This study also employed a widely used high-fat diet-induced obesity murine model [27] to facilitate investigation. As mentioned above, ER stress mediates the effects of obesity on aggravating sepsis-induced organ injury. Moreover, inflammation and oxidation are crucial mechanisms that mediate sepsis-induced acute lung injury in obesity [3,30]. The role of exosomes from MSCs in modulating ER stress, inflammation, and oxidation in lung tissues of lipopolysaccharide-stimulated obese mice was also investigated in this study.

## 2. Materials and Methods

### 2.1. Characterization of Exosomes

This study employed exosomes from human placenta choriodecidual membrane-derived MSCs (pcMSCs) for investigations. The Joint Institutional Review Board of Taipei Medical University, Taipei, Taiwan, approved the human study regarding acquisition of human placenta and isolation of human pcMSCs (N202101014). Preparation of human pcMSCs and exosomes were performed, as we have recently reported [27,31]. Following that, exosomes were extracted from human pcMSCs by culture medium harvesting, centrifugation, supernatant collection, filtering, and ultracentrifugation (Beckman Coulter Optima L-80XP Ultracentrifuge; 100,000× *g*; 4 °C; 90 min; Type 50.2 Ti rotor, k-factor: 157.7; Beckman Coulter Inc., Brea, CA, USA) [27]. The pellets were then resuspended, pooled, ultracentrifuged, resuspended, purified, and ultracentrifuged once more. The gradient’s top fractions were collected, diluted, and centrifuged. The pellets were reconstituted and kept at −80 °C. Exosome particles and sizing were analyzed (NanoSight NS300; Malvern Panalytical, Malvern, UK) [32] and their morphology was estimated using TEM, markers CD63 and CD9 were detected using immunoblotting assay [33]. The production yield of isolated exosomes from one 10-cm dish seeding 1 × 10^6^ human pcMSCs was approximate 4.2~10.0 × 10^8^ particles.

### 2.2. TEM Analysis

To examine the morphology of the exosomes using TEM, an exosome suspension was fixed and transferred to grids (Polysciences, Warrington, PA, USA) and dried. The morphology of exosomes was observed using a transmission electron microscope (Hitachi HT-7700; Hitachi, Tokyo, Japan) [33].

### 2.3. Animals, Study Approval, and Diet-Induced Obesity Murine Model

The Institutional Animal Use and Care Committee of Taipei Medical University approved all animal studies (LAC-2017-0454). Adult male C57BL/6J mice (National Laboratory Animal Center, Taipei, Taiwan) were acquired at 7 weeks of age and utilized in this study. Mice were fed standard laboratory diet, given access to unrestricted water, and subjected to a 12 h light/12 h dark cycle. The mice were handled and cared for in compliance with National Institutes of Health (NIH) guidelines, USA. After housing for 7 days, all mice received a 12-week feeding of high-fat diet (60% kcal from fat, 20% kcal from carbohydrates, and 20% kcal from protein; Research Diets, New Brunswick, NJ, USA) for diet-induced obesity (DIO) [27]. Figure S1 in the supplementary materials demonstrated the effects of this model on inducing obesity in mice.

### 2.4. Biodistribution

The exosomes (1 × 10^8^ particles per mouse) were conjugated with Cy7 mono NHS ester (Amersham Biosciences, Buckinghamshire, UK) and intraperitoneally (i.p.) injected into DIO mice. After exosomes administration mice were euthanized at 0, 2, 24, or 48 h and their organs (heart, lungs, liver, kidney, and spleen) were harvested for bioluminescence imaging assay and analysis (IVIS Lumina XRMS and Living Image software, both from PerkinElmer, Waltham, MA, USA) [27].

### 2.5. Experimental Design

The DIO mice were randomized into four groups. The negative control group (the DIO group) received injection of normal saline (0.5 mL, i.p.). The positive control group (the DIOLPS group) received lipopolysaccharide (10 mg/kg, i.p.; *Escherichia coli* 0127:B8, Sigma-Aldrich, Burlington, MA, USA). The treatment control group (the DIOExo group) received exosome (1 × 10^8^ particle per mouse, i.p.). The experimental group (the DIOLPSExo group) received lipopolysaccharide (10 mg/kg, i.p.) and exosomes (1 × 10^8^ particle per mouse, i.p.). Exosomes were administrated at 2 h after normal saline or lipopolysaccharide.

All mice were closely monitored for 48 h after normal saline or lipopolysaccharide injection to determine the 48-h survival rate of each group. The doses of lipopolysaccharide and exosomes were determined according to our recent report [27]. The levels of pulmonary ER stress, inflammation, oxidation, apoptosis, and lung injury in the surviving mice were then measured.

### 2.6. Lung Function

The mice received zoletil/xylazine (40/10 mg/kg, i.p.) for anesthesia, then tracheostomy followed by tracheostomy tube insertion was performed. Then, the anesthetized mice were mechanical ventilated with a computerized small animal ventilator (Finepoint; Buxco Electronic, Wilmington, NC, USA). The ventilation rate and tidal volume were set at 150 breaths/min and 0.2 mL, respectively. Tidal volume, peak inspiratory flow, end expiratory volume, and airway resistance were recorded using a FinePointe™ RC System (Buxco Research Systems; New Brighton, MN, USA), as previously reported [34].

### 2.7. Bronchoalveolar Lavage, Lung Harvesting, and Wet/Dry Weight (W/D) Ratio Determination

Through the tracheostomy tube, the anesthetized mice received 5 times of bronchoalveolar lavage with 1 mL sterile normal saline and the bronchoalveolar lavage fluid (BALF) samples were collected. With 1:1 dilution with trypan blue dye (Sigma-Aldrich), the total cell number in BALF was measured, as we have previously reported [35]. Then, decapitation was performed to euthanize the mice. The lung tissues were then collected. A portion of the lung tissues were promptly frozen using liquid nitrogen and kept at −80 °C for subsequent examination. Furthermore, the wet/dry weight ratio (i.e., tissue water content) was determined using a procedure that we have previously reported [27]. In summary, newly obtained lung tissues were weighed and then put in an oven set at 80 °C. They were weighed again after 24 h, and the wet/dry weight ratio data were computed.

### 2.8. Lung Histology

For histological investigation, lung tissues were isolated immediately after euthanasia. For hematoxylin and eosin staining a portion of the lung tissues were fixed in 10% formalin (Sigma-Aldrich). To determine the extent of lung injury, morphological characteristics (including polymorphonuclear neutrophils (PMN) infiltration, focal necrosis, and hemorrhages/congestion) were evaluated under a light microscope at 200× magnification, and lung injury score (i.e., the sum of each independent variable; neutrophils in the alveolar space (0: none; 2: >5), neutrophils in the interstitial space (0: none; 2: >5), hyaline membranes (0: none; 2: >1), proteinaceous debris filling the airspaces (0: none; 2: >1), alveolar septal thickening (0: <2×; 2: >4×), and normalized to the number of fields evaluated) were calculated to determine lung injury levels [36].

### 2.9. Immunoblotting Assay

Exosome samples were prepared, as we have previously reported [27]. Snap-frozen lung tissues were processed also as we have previously reported [35]. Equal amounts of proteins (40 μg) from exosome samples and lung tissue samples were then separated by electrophoresis and then transferred to nitrocellulose membranes (Bio-Rad Laboratories, Hercules, CA, USA). For exosome samples, the membranes were incubated with one of the primary antibodies against exosome markers, including CD9 (anti-CD9 antibody, 20597-1-AP; Proteintech, Rosemont, IL, USA) and CD63 (anti-CD63 antibody, 25682-1-AP; Proteintech) [33]. For lung tissue samples, the membranes were incubated with one of the primary antibodies against ER stress related proteins, including GRP78 (anti-GRP78 antibody, ab21685; Abcam, Cambridge, UK) [5], phosphorylated eIF2α (p-eIF2α) (anti-p-eIF2α Ser51 antibody, #9721; Cell Signaling Technology, Danvers, MA, USA) [37], and CHOP (anti-CHOP L63F7 antibody, #2895; Cell Signaling) [37]. For lung tissue samples, the membranes were also incubated with one of the primary antibodies against transcription factors, including nuclear factor-κB (NF-κB) (anti-phosphorylated NF-κB p65 Ser536 antibody, #3033, Cell Signaling) [17] and hypoxia-inducible facror-1α (HIF-1α) (anti-HIF-1α antibody, IR113-466, iReal Technology, Hsinchu, Taiwan) [17]. For comparison, the membranes of lung tissue samples were also incubated with the primary antibody of the internal standard Actin (anti-Actin antibody, A5441; Sigma-Aldrich) [37]. Bound antibody was detected through chemiluminescence (ECL Plus kit; Amersham). Protein band density was measured using densitometry (ImageJ). Figure S2 in the supplementary materials demonstrated the original representative gel photography of immunoblotting assays. 

### 2.10. Enzyme-Linked Immunosorbent Assay (ELISA)

The freshly frozen lung tissues were processed as we have previously reported [35]. The supernatants were obtained after homogenizing and centrifuging freshly frozen lung tissues. The pulmonary concentrations of cytokines (including tumor necrosis factor-α (TNF-α), interleukin-1β (IL-1β), and IL-6) and leptin were assayed using ELISA kits (all kit assays were from Enzo Life Science, Farmingdale, NY, USA).

### 2.11. Immunohistochemistry Staining

The formalin-fixed lung tissue samples were processed, as above-mentioned. The tissue sections were then incubated with the primary antibody against macrophage activation (i.e., M1 phase polarization) related protein inducible nitric oxide synthase (iNOS) (anti-iNOS antibody, ab3523; Abcam), NLR family pyrin domain containing 3 (NLRP3) inflammasomes (anti-NLRP3 antibody, ab263899; Abcam), or malondialdehyde (marker for lipid peroxidation) (anti-malondialdehyde antibody, ab27642, Abcam) [27]. All sections were observed (TissueGnostics Axio Observer Z1 microscope; TissueGnostics, Vienna, Austria) and analyzed (Image J).

### 2.12. Terminal Deoxynucleotidyl Transferase dUTP Nick End (TUNEL) Assay

The TUNEL technique was used to assess the main feature of apoptosis, DNA fragmentation, in lung tissues [37]. An in situ cell death detection kit (Roche, Indianapolis, IN, USA) was employed, and the labeling of the apoptotic cells in lung tissues was carried out in accordance with the manufacturer’s instructions. Staining with DAPI (Sigma-Aldrich) was performed to detect total nuclei. All sections were scanned and examined as previously reported [37], and the mean TUNEL-positive cell count in each group was calculated using five randomly selected fields (0.25 mm^2^).

### 2.13. Statistical Analysis

Data were presented as mean ± standard deviations. Between-group differences were analyzed using one-way analysis of variance and post hoc pairwise comparisons with Tukey’s test. The Kaplan–Meier analysis was performed to analyze the 48 h survival rates. A *p* value of <0.05 was considered as statistically significant.

## 3. Results

### 3.1. Confirmation and Biodistribution of Exosomes

**Figure 1:**
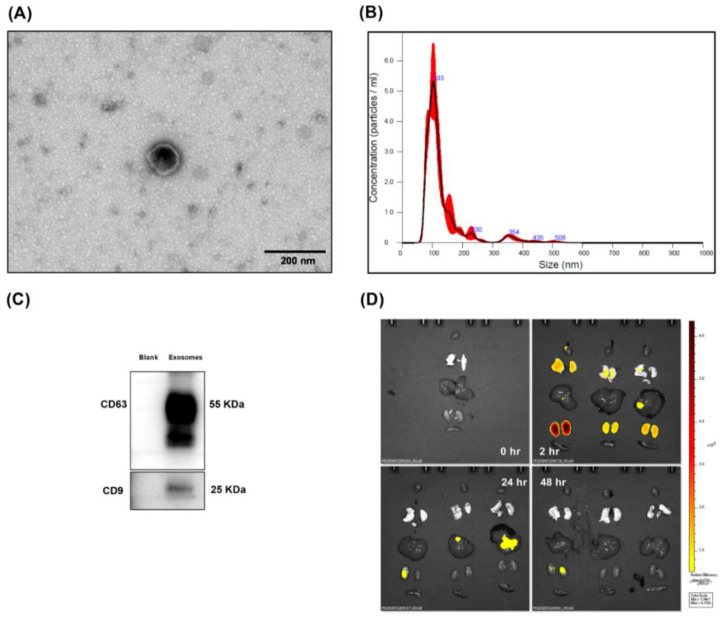
(**A**) Representative transmission electron microscopic images of exosomes (from human placenta choriodecidual membrane-derived mesenchymal stem cells). (**B**) Particles and sizing of exosomes. (**C**) Representative gel photography of exosomes markers CD63 and CD9, using immunoblotting assay. (**D**) Biodistribution of exosomes conjugated with Cy7 mono NHS ester (1 × 10^8^ particles per mouse) in the heart, lungs, liver, kidney, and spleen of diet-induced obese mice, using ex vivo bioluminescence imaging assay, measured at 0, 2, 24, and 48 h after intraperitoneal administration.

The presence of exosomes was established by observing a cup-shaped morphology using TEM, as illustrated in Figure 1A. Our data also showed that the particle size of exosomes was approximate 100–150 nm, using the nanoparticle tracking analysis (Figure 1B). Positive markers CD63 and CD9 of exosomes were also observed, using immunoblotting assay (Figure 1C). Moreover, bioluminescence imaging assay illustrated significant signal of Cy7-conjugated exosomes in the heart, lungs, liver, and kidney in the DIO mice, measured at 2 and 24 h, but not at 48 h, after administration (Figure 1D). Notably, bioluminescence imaging assay illustrated no significant signal of Cy7-conjugated exosomes in spleen in the DIO mice measured at 2, 24, and 48 h after administration.

### 3.2. Survivorship

**Figure 2:**
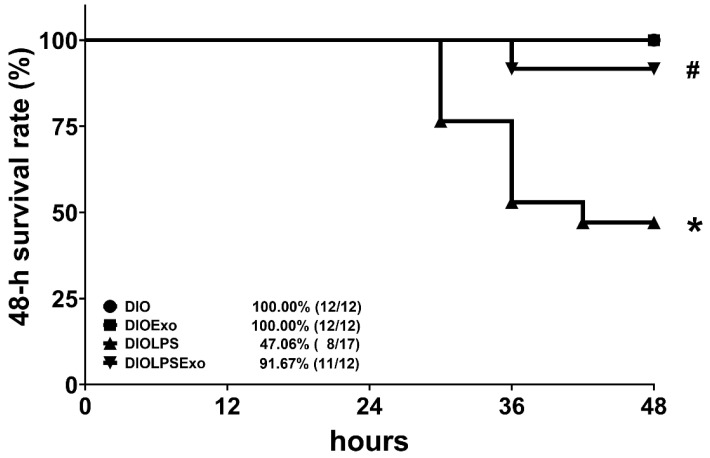
The 48 h (48-h) survival rates, as determined by calculating the number of mice survived the 48-h observational duration in each group after normal saline or lipopolysaccharide administration. A total of 12, 12, 17, and 12 mice in the DIO, the DIOExo, the DIOLPS, and the DIOLPSEXo groups were employed, respectively, for this assay. DIO: the diet-induced obese mice plus intraperitoneal (i.p.) administration of normal saline (0.5 mL) group. DIOExo: the diet-induced obese mice plus exosomes (from human placenta choriodecidual membrane-derived mesenchymal stem cells, 1 × 10^8^ particle per mouse, i.p.) group. DIOLPS: the diet-induced obese mice plus lipopolysaccharide (10 mg/kg, i.p.) group. DIOLPSExo: the diet-induced obese mice plus lipopolysaccharide plus exosomes group. * *p* < 0.05, versus the DIO group. # *p* < 0.05, the DIOLPSExo group versus the DIOLPS group.

Figure 2 illustrates the 48 h survival rates. All mice in the DIO group survived the experiment. The 48-h survival rate in the DIO group was 100%. Notably, lipopolysaccharide induces significant mortality in DIO mice, as the 48-h survival rate in the DIOLPS group was 47.1%. Statistical analysis revealed that the 48-h survival rate in the DIOLPS group was significantly lower than that in the DIO group (*p* = 0.003). Exosomes mitigate the adverse effect of lipopolysaccharide, as the 48-h survival rate in the DIOLPSExo group (91.7%) was significantly higher than that in the DIOLPS group (*p* = 0.015). Moreover, all mice in the DIOExo group survived the experiment. The 48-h survival rate in the DIOExo group was 100%, indicating that exosomes per se exert no significant effects on mortality in DIO mice.

### 3.3. Lung Injury

**Figure 3:**
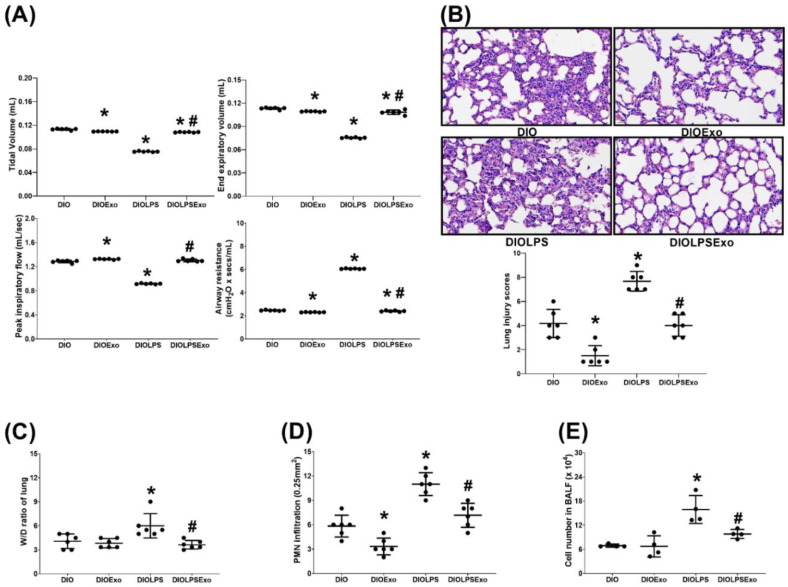
(**A**) Lung function, representing tidal volume, peak inspiratory flow, end expiratory volume, and airway resistance. Data were obtained from 6 mice from each group for each parameter. (**B**) Representative microscopic findings of the lung tissues stained with hematoxylin-eosin (200×) and the organ injury scores. Data were obtained from 6 mice in each group. (**C**) Wet/dry weight ratio (W/D ratio, indicator of tissue water content) of the lung tissues. Data were obtained from 6 mice in each group. (**D**) The levels of, polymorphonuclear leukocyte (PMN) filtration in lung tissues. Data were obtained from 6 mice in each group. (**E**) The cell number in bronchoalveolar lavage fluid (BALF) in lung tissues. Data were obtained from 6 mice in each group. All assays were measured at 48 h after normal saline or lipopolysaccharide administration. Data represented as mean ± standard deviations. DIO: the diet-induced obese mice plus intraperitoneal (i.p.) administration of normal saline (0.5 mL) group. DIOExo: the diet-induced obese mice plus exosomes (from human placenta choriodecidual membrane-derived mesenchymal stem cells, 1 × 10^8^ particle per mouse, i.p.) group. DIOLPS: the diet-induced obese mice plus lipopolysaccharide (10 mg/kg, i.p.) group. DIOLPSExo: the diet-induced obese mice plus lipopolysaccharide plus exosomes group. * *p* < 0.05, versus the DIO group. # *p* < 0.05, the DIOLPSExo group versus the DIOLPS group.

Figure 3A illustrates data of the lung function assay. Lipopolysaccharide induces significantly adverse impacts on lung function in DIO mice. The tidal volume, peak inspiratory flow, and end expiratory volume in the DIOLPS group were significantly lower than those in the DIO group (all *p* < 0.001). By contrast, the airway resistance in the DIOLPS group was significantly higher than that in the DIO group (*p* < 0.0001). Notably, exosomes mitigate the adverse effects of lipopolysaccharide. The tidal volume, peak inspiratory flow, and end expiratory volume in the DIOLPSExo group were significantly higher than those in the DIOLPS group (all *p* < 0.001). The airway resistance, by contrast, in the DIOLPSExo group was significantly lower than that in the DIOLPS group (*p* < 0.001).

Figure 3B illustrates histological analysis of lung tissues and lung injury scores. Lipopolysaccharide induces significantly PMN infiltration, focal necrosis, and hemorrhages/congestion in lung tissues in DIO mice and the lung injury score in the DIOLPS group was significantly higher than that in the DIO group (*p* < 0.001). Notably, exosomes mitigate the adverse effects of lipopolysaccharide, as the lung injury score in the DIOLPSExo group was significantly lower than that in the DIOLPS group (*p* < 0.001). Moreover, data of the W/D ratio in lung tissues (Figure 3C), PMN infiltration level in lung tissues (Figure 3D), and cell number in BALF (Figure 3E) parallel those of the lung injury score (Figure 3B).

The tidal volume, end expiratory volume, and airway resistance in the DIOExo group were significantly lower than those in the DIO group (*p* < 0.001, =0.002, and <0.001, respectively; Figure 3A), whereas the peak inspiratory flow in the DIOExo group was significantly higher than that in the DIO group (*p* = 0.003; Figure 3A). Moreover, the lung injury score (*p* < 0.001; Figure 3B) and the PMN infiltration level (*p* = 0.018; Figure 3D) in the DIOExo group were significantly lower than those in the DIO group, whereas the W/D ratio (Figure 3C) and cell number in BALF (Figure 3E) in the DIOExo and the DIO groups were not significantly different. These data indicate that exosomes per se may exert significant effects on improving lung function and mitigating lung injury in DIO mice.

### 3.4. ER Stress

**Figure 4:**
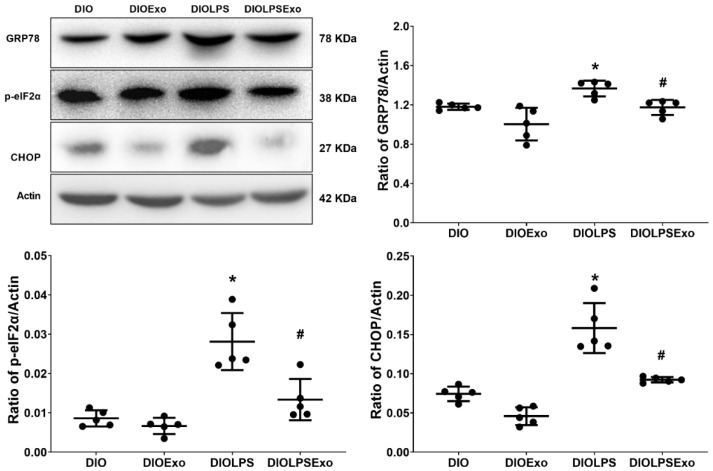
Representative gel photography of endoplasmic reticulum stress related proteins, including glucose-regulated protein 78 (GRP78), phosphorylated eukaryotic initiation factor 2α (p-eIF2α), and C/EBP homologous protein (CHOP), and the internal standard Actin assayed using immunoblotting assay and the relative band density of GRP78/Actin, p-eIF2α/Actin, and CHOP/Actin ratios in lung tissues. Data were obtained from 5 mice from each group for each parameter. All assays were measured at 48 h after normal saline or lipopolysaccharide administration. Data represented as mean ± standard deviations. DIO: the diet-induced obese mice plus intraperitoneal (i.p.) administration of normal saline (0.5 mL) group. DIOExo: the diet-induced obese mice plus exosomes (from human placenta choriodecidual membrane-derived mesenchymal stem cells, 1 × 10^8^ particle per mouse, i.p.) group. DIOLPS: the diet-induced obese mice plus lipopolysaccharide (10 mg/kg, i.p.) group. DIOLPSExo: the diet-induced obese mice plus lipopolysaccharide plus exosomes group. * *p* < 0.05, versus the DIO group. # *p* < 0.05, the DIOLPSExo group versus the DIOLPS group.

Figure 4 illustrates data of the expression levels of GRP78, p-eIF2α, and CHOP using immunoblotting assay to demonstrate ER stress in lung tissues. Lipopolysaccharide induces ER stress in lung tissues in DIO mice. The expression levels of GRP78, p-eIF2α, and CHOP in the DIOLPS group were significantly higher than those in the DIO group (*p* = 0.048, <0.001, and <0.001, respectively). Notably, exosomes mitigate the adverse effects of lipopolysaccharide. The expression levels of GRP78, p-eIF2α, and CHOP in the DIOLPSExo group were significantly lower than those in the DIOLPS group (*p* = 0.038, <0.001, and <0.001, respectively). Moreover, the expression levels of GRP78, p-eIF2α, and CHOP in the DIO and the DIOExo groups were not significantly different, indicating that the exosomes exert no significant effects on ER stress in lung tissues in DIO mice.

### 3.5. Lung Inflammation

**Figure 5:**
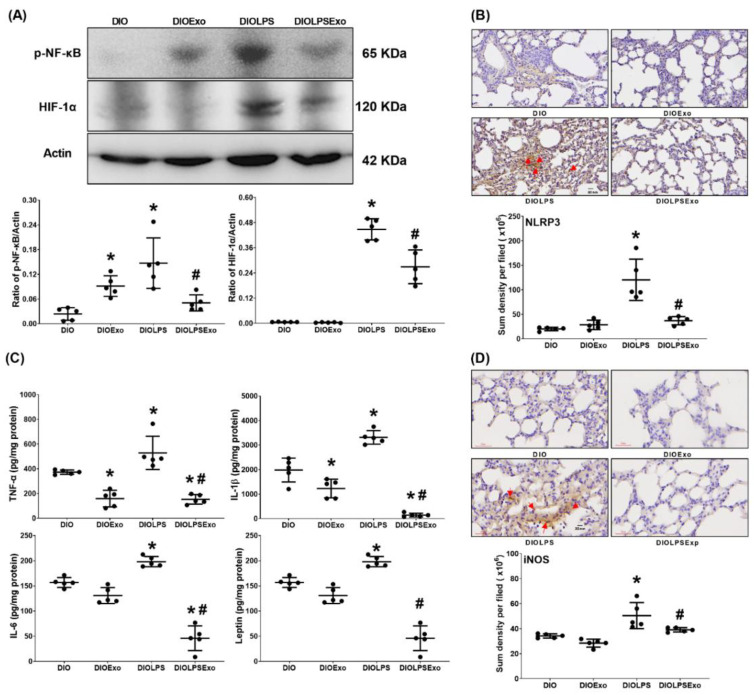
(**A**) Representative gel photography of phosphorylated nuclear factor-kB (p-NF-kB), hypoxia-inducible factor-1α (HIF-1α), and Actin (internal standard), assayed using immunoblotting assay and the relative band density of p-NF-kB/Actin, HIF-1α/Actin ratios in lung tissues. Data were obtained from 5 mice in each group. (**B**) Representative microscopic images of NLR family pyrin domain containing 3 (NLRP3; marked by red arrow), indicator of inflammasomes; using immunohistochemistry staining assay and the quantitative sum intensities of in lung tissues. Data were obtained from 5 mice in each group. (**C**) The concentrations of tumor necrosis factor-α (TNF-α), interleukin 1-β (IL-1β), IL-6, and leptin in lung tissues, analyzed using enzyme-linked immunosorbent assay. Data were obtained from 6 mice in each group. (**D**) Representative microscopic images of inducible nitric oxide synthase (iNOS, indicator of pro-inflammatory M1 phase macrophage polarization; marked by the red arrow) and the quantitative sum intensities of in lung tissues. Data were obtained from 5 mice in each group. All assays were performed at 48 h after normal saline or lipopolysaccharide administration. Data represented as mean ± standard deviations. DIO: the diet-induced obese mice plus intraperitoneal (i.p.) administration of normal saline (0.5 mL) group. DIOExo: the diet-induced obese mice plus exosomes (from human placenta choriodecidual membrane-derived mesenchymal stem cells, 1 × 10^8^ particle per mouse, i.p.) group. DIOLPS: the diet-induced obese mice plus lipopolysaccharide (10 mg/kg, i.p.) group. DIOLPSExo: the diet-induced obese mice plus lipopolysaccharide plus exosomes group. * *p* < 0.05, versus the DIO group. # *p* < 0.05, the DIOLPSExo group versus the DIOLPS group.

Figure 5A illustrates the expression levels of the transcription factors NF-κB and HIF-1α in lung tissues using immunoblotting assay. Lipopolysaccharide significantly activates NF-κB and HIF-1α. The expression levels of p-NF-κB and HIF-1α in lung tissues in the DIOLPS group were significantly higher than those in DIO group (both *p* < 0.001). Notably, exosomes mitigate the effects of lipopolysaccharide, as the expression levels of p-NF-κB and HIF-1α in lung tissues in the DIOLPSExo group were significantly lower than those in the DIOLPS group (*p* = 0.003 and <0.001, respectively). Similar picture was observed in the expression levels of NLRP3 (indicator of inflammasomes; Figure 5B), TNF-α, IL-1β, IL-6, and leptin (Figure 5C), and iNOS (indicator of pro-inflammatory M1 phase macrophage polarization; Figure 5D).

Moreover, the expression levels of p-NF-κB in the DIOExo group was significantly higher than that in the DIO group (*p* = 0.036; Figure 5A), whereas the expression levels of TNF-α and IL-1β in the DIOExo group were significantly lower than those in the DIO group (*p* = 0.003 and =0.015, respectively; Figure 5C). In addition, the expression levels of HIF-1α, NLRP3, IL-6, leptin, and iNOS in lung tissues in the DIO and the DIOExo groups were not significantly different. These data indicate that exosomes per se may exert certain effects on mitigating lung inflammation in DIO mice.

### 3.6. Lung Oxidation and Apoptosis

**Figure 6:**
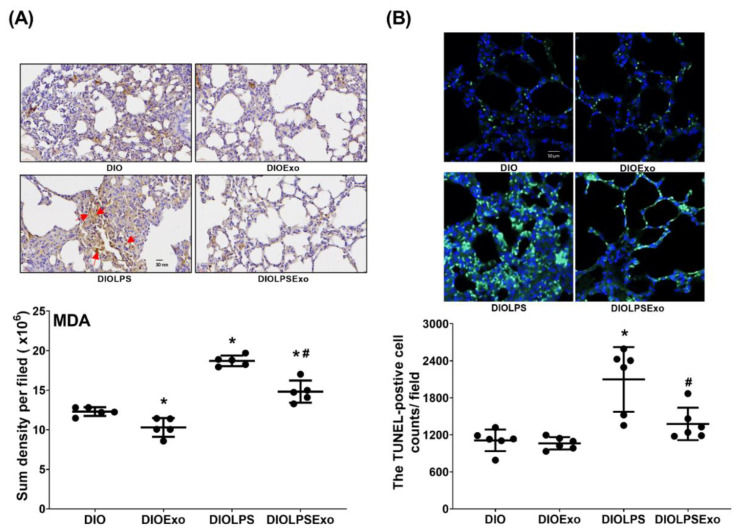
(**A**) Representative microscopic images of malondialdehyde (MDA, indicator of lipid peroxidation, using immunohistochemistry staining assay; marked by the red arrow) and the quantitative sum intensities of MDA in lung tissues. Data were obtained from 5 mice in each group. (**B**) Representative DNA fragmentation microscopic images (indicator of apoptosis, marked by the green fluorescence dots, 200×) in lung tissues assayed using the terminal deoxynucleotidyl transferase dUTP nick end labeling (TUNEL) method and the count of TUNEL-positive cells (0.25 mm^2^). Data were obtained from 6 mice in each group. All assays were measured at 48 h after normal saline or lipopolysaccharide administration. Data represented as mean ± standard deviations. DIO: the diet-induced obese mice plus intraperitoneal (i.p.) administration of normal saline (0.5 mL) group. DIOExo: the diet-induced obese mice plus exosomes (from human placenta choriodecidual membrane-derived mesenchymal stem cells, 1 × 10^8^ particle per mouse, i.p.) group. DIOLPS: the diet-induced obese mice plus lipopolysaccharide (10 mg/kg, i.p.) group. DIOLPSExo: the diet-induced obese mice plus lipopolysaccharide plus exosomes group. * *p* < 0.05, versus the DIO group. # *p* < 0.05, the DIOLPSExo group versus the DIOLPS group.

Figure 6A illustrates lung oxidation status determined by assaying the expression level of malondialdehyde (the indicator of lipid peroxidation) in lung tissues using immunohistochemistry staining assay. Presence of lung apoptosis was determined by assaying DNA fragmentation using TUNEL assay (Figure 6B). Lipopolysaccharide significantly induces lipid peroxidation and apoptosis in DIO mice. The expression level of malondialdehyde and the TUNEL-positive cell count in lung tissues in the DIOLPS group was significantly higher than those in the DIO group (both *p* < 0.001). Exosomes significantly mitigate the effects of lipopolysaccharide, as the expression level of malondialdehyde and the TUNEL-positive cell count in the DIOLPSExo group were significantly lower than those in the DIOLPS group (*p* < 0.001 and =0.003, respectively). Moreover, the expression level of malondialdehyde in lung tissues in the DIOExo group was significantly lower than that in the DIO group (*p* = 0.030), whereas the TUNEL-positive cell count in the DIOExo and the DIO groups were not significantly different. These data indicate that exosomes per se exert certain effects on mitigating oxidation in DIO mice.

## 4. Discussion

The present study revealed that lipopolysaccharide (produced by Gram-negative bacteria) increases the mortality rate and causes acute lung injury in diet-induced obese mice. Our results indicated that exosomes obtained from human pcMSCs can counteract the damaging effects of lipopolysaccharide, and they improve the survival rate and reduce lung injury in lipopolysaccharide-stimulated obese mice. Moreover, the findings of the present study demonstrated the effects of lipopolysaccharide on the upregulation of ER stress, inflammation, oxidation, and apoptosis in lung tissues in obese mice. Notably, our data demonstrated that exosomes from human pcMSCs also counteract the adverse effects of lipopolysaccharide and mitigate ER stress, inflammation, oxidation, and apoptosis in lung tissues in lipopolysaccharide-stimulated obese mice. Our findings collectively supported our hypothesis and indicated that exosomes from human pcMSCs are potential therapeutics against sepsis in obesity. Given the lack of an effective sepsis-induced acute lung injury therapy in obesity, the findings of the present study provided direct evidence supporting the use of exosomes from human pcMSCs as a novel and effective treatment modality for sepsis-induced acute lung injury therapy in obesity.

According to findings from this study, lipopolysaccharide treatment led to a mortality rate of more than 50% among obese mice. In another recent study that we conducted, we used a sepsis murine model identical to the one used in the present study and reported results that were similar to those of the present study [27]. Moreover, our findings verified that lipopolysaccharide can readily cause acute lung injury in obese mice. On the basis of the same sepsis model used in the previous study that we conducted, we discovered that lipopolysaccharide induces acute liver injury in obese mice. These findings highlight the damaging role of lipopolysaccharide (produced by Gram-negative bacteria) in inducing multiple organ injury/failure in obese mice. Because multiple organ failure is a major cause of death in severe sepsis [38], significant mortality observed in the present study is reasonably consistent with the findings of our previous report [27]. Data from the present study, in concert with those from our recent report [27], collectively demonstrated the therapeutic effects of exosomes from human pcMSCs on mitigating lipopolysaccharide-induced organ injury (e.g., lung and liver) in obese mice. Given the aforementioned effects, exosomes from human pcMSCs can be inferred to improve the survivorship of obese mice that are exposed to lipopolysaccharide.

After mice are exposed to lipopolysaccharide, NF-kB acts to directly transcribe several inflammatory genes (e.g., TNF-α, IL-1β, IL-6, and leptin), and this process plays a key role in the pathogenesis of several inflammatory disease processes [39,40,41]. A study reported a continuous increase in pulmonary cytokine levels (especially TNF-α) and oxidative stress levels in non-obese and diet-induced obese groups after lipopolysaccharide administration [42]. HIF-1α signaling is also involved in lipopolysaccharide-induced lung inflammation, because HIF-1α plays a crucial role in immune and inflammatory reactions and increases the expression of several genes (e.g., those for vascular endothelial growth factor, erythropoietin, and several glycolytic enzymes) in response to hypoxia [43,44]. In addition, HIF-1α activation leads to M1 phase macrophage polarization and subsequent macrophage activation [27]. Inflammasomes, especially the most studied NLRP3 inflammasomes, have been reported to be activated by sepsis-induced acute lung injury, ventilator-induced lung injury, asthma, chronic obstructive pulmonary disease, and other respiratory diseases [45]. Notably, in the present study, we observed upregulated expression levels of NF-kB, HIF-1α, NLRP3 inflammasomes, cytokines, leptin, iNOS (i.e., indicator of M1 phase macrophage polarization), and oxidative stress (lipid peroxidation) in the lungs of lipopolysaccharide-stimulated obese mice. The occurrence of inflammation in concert with oxidation can readily induce apoptosis [37]; thus, the increased occurrence of apoptosis in the lungs of lipopolysaccharide-stimulated obese mice was an expected finding in the present study. Moreover, the findings of the present study demonstrated the potent role of exosomes from human pcMSCs in inhibiting the production of cytokines, leptin, and malondialdehyde (a marker of lipid peroxidation) and in negatively regulating the activation of NF-kB, HIF-1α, and NLRP3 inflammasomes in the lungs of lipopolysaccharide-stimulated obese mice. Therefore, the role of exosomes from human pcMSCs in inhibiting apoptosis in the aforementioned lipopolysaccharide-stimulated obese mice was also an expected finding.

Studies have demonstrated the role of sepsis in inducing ER stress [7,8,9,46]. A recent study revealed that ER stress is involved in the development of acute lung injury induced by lipopolysaccharide administration in vivo and enhanced in lipopolysaccharide-stimulated airway epithelial cells in vitro [47]. A murine model of lipopolysaccharide-induced lung injury indicated that the mRNA and protein levels of the UPR markers GRP78, p-eIF2α, and CHOP were significantly elevated in lung tissues [48,49]. The present study also verified that lipopolysaccharide induces ER stress; specifically, we detected the significant upregulation of GRP78, p-eIF2α, and CHOP expression levels in the lungs of lipopolysaccharide-stimulated obese mice. Moreover, we provided clear evidence of the potent role of exosomes from human pcMSCs in reducing ER stress and negatively regulating the increased expression levels of GRP78, p-eIF2α, and CHOP in the lungs of lipopolysaccharide-stimulated obese mice. Because the upregulation of ER stress-related proteins (especially CHOP) can also activate apoptosis [48,49], we thus inferred that the mechanisms underlying the inhibitory effects of exosomes from human pcMSCs on apoptosis are linked to their effects on mitigating lipopolysaccharide-induced ER stress in obese mice.

Studies have indicated that lipopolysaccharide induces inflammation and causes ER stress in vivo [17,46,48]. Additionally, a study suggested an interaction between inflammation and ER stress, which may further complicate sepsis [50]. The interaction between inflammation and ER stress involves complex crosstalk loops [51,52,53], and a main crosstalk loop operates between the UPR and NF-kB through secreted cytokines (e.g., TNFα and IL-1β) and Toll-like receptors. [51,52,53]. Another crosstalk loop between the UPR and HIF-1α has also been reported [54,55,56]. The findings of the present study provided clear evidence to demonstrate that exosomes from human pcMSCs can inhibit both inflammation and ER stress. Moreover, given the role of exosomes from human pcMSCs in mitigating cytokine production, they are likely to have significant effects on the blocking of the interaction between inflammation and ER stress. Therefore, we further conjecture that the above-mentioned effect contributes to the therapeutic effects of exosomes from human pcMSCs on sepsis observed in the present study.

**Figure 7:**
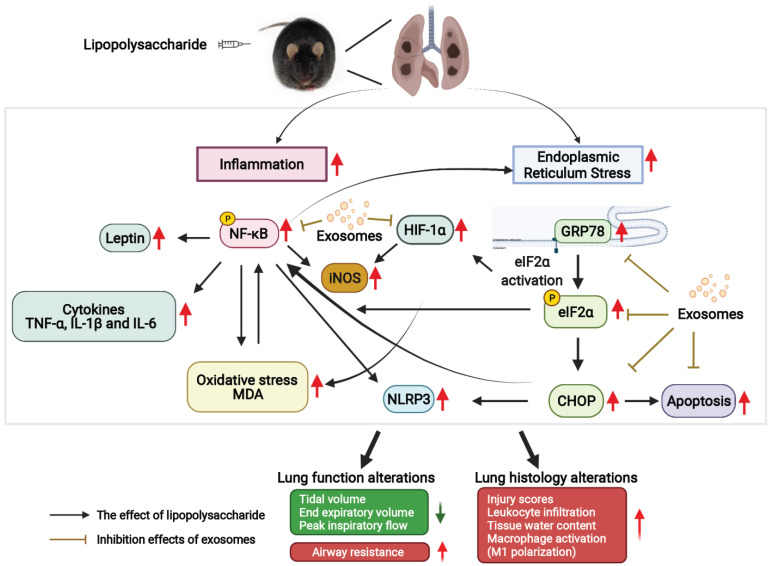
The diagram summarizing the effects of exosomes (from human placenta choriodecidual membrane-derived mesenchymal stem cells) on mitigating lung injury and the mechanisms (including endoplasmic reticulum stress, inflammation, oxidation, and apoptosis in lung tissues) in lipopolysaccharide-treated diet-induced obese mice, measured at 48 h after lipopolysaccharide administration. CHOP: C/EBP homologous protein. p-eIF2α: phosphorylated eukaryotic initiation factor 2α. GRP78: glucose-regulated protein. HIF-1α: hypoxia-inducible factor-1α. IL-1β: interleukin 1β. IL-6: interleukin-6. iNOS: inducible nitric oxide synthase. MDA: malondialdehyde. p-NF-kB: phosphorylated nuclear factor-kB. NLRP3: NLR family pyrin domain containing 3. TNF-α: tumor necrosis factor-α.

Figure 7 presents the effects and mechanisms of exosomes from human pcMSCs in relation to lipopolysaccharide-induced lung injury in obese mice.

Data from the present study, in concert with those from our recent report [27], provided clear evidence to demonstrate the potent therapeutic effects of exosomes from human pcMSCs against sepsis in obesity. Sepsis in obesity is a critical condition associated with complex mechanisms, including ER stress, inflammation, oxidative stress, and apoptosis [3,18,19,30]. Data from the present study further demonstrated that the mechanisms underlying the therapeutic effects of exosomes from human pcMSCs may involve their effects on mitigating ER stress, inflammation, oxidative stress, and apoptosis. As mentioned above, the therapeutic effects of exosomes mainly involve transferal of the functional cargos from MSCs to the recipient cells [23,24]. Through membrane fusion or endocytosis, the components of exosomes (e.g., proteins, peptides, lipids, cytokines, mRNAs, and/or miRNAs) will release to the cytosol and result in alterations of functional and/or phenotypical expression of the recipient cells, through modifying certain gene expression and signaling pathways [23,24]. This concept is confirmed by the findings from our recent study that let-7i-5p miRNA mediates the effects of exosomes from human pcMSCs on mitigating sepsis-induced liver injury in obese mice [27]. Considering that overexpression of let-7i-5p miRNA can inhibit NF-κB activation, mitochondrial dysfunction and apoptosis, as previously demonstrated in a cell model of myocardial ischemia in cardiomyocytes [57], we conjecture that the therapeutic effects of exosomes observed in this study may very likely be mediated by let-7i-5p miRNA. More studies are needed before further conclusion can be drawn.

Similar to sepsis in obesity, the complex mechanisms of ER stress, inflammation, oxidative stress, and apoptosis are also crucial mechanisms in mediating the critical condition of infection with severe acute respiratory syndrome coronavirus 2 (SARS-CoV-2) [58]. Because exosomes from human pcMSCs, as demonstrated in the present study, can mitigate ER stress, inflammation, oxidation, and apoptosis, we thus further conjecture that exosomes from human pcMSCs may exert certain beneficial effects against SARS-CoV-2 infection. This concept is supported by previous data that exosomes from bone marrow-derived MSCs can restore oxygenation, downregulate cytokine storm, and reconstitute immunity in SARS-CoV-2 infection patients [59]. Similar mechanisms also contribute to the development of acute and chronic inflammatory diseases, including rheumatoid arthritis, juvenile rheumatoid arthritis, inflammatory bowel disease, ankylosing spondylitis, psoriasis, psoriatic arthritis, graft-versus-host disease, diabetic nephropathy, organ fibrosis, etc. [60,61,62]. Previous data also demonstrated the therapeutic potentials of exosomes from MSCs against inflammatory diseases [63,64]. Based on these data, we further conjecture that exosomes from human pcMSCs may exert beneficial effects against these above-mentioned inflammatory diseases. More studies are needed before further conclusion can be drawn.

Studies have reported that obesity is associated with low-grade inflammation [65,66].

The mechanisms, as mentioned above, involve the obesity-induced expansion of adipose tissue which results in the activation of various intrinsic signaling pathways (e.g., adipocyte death, hypoxia, and mechanical stress) that can trigger inflammation [4,67]. A consistent link exists between the low-grade chronic inflammation of adipose tissue and the infiltration of proinflammatory macrophages and other immune cells that secrete proinflammatory cytokines in obesity [4,67,68,69]. Moreover, enhanced profiles of ER stress and oxidative stress have been reported in obesity [18,19,70]. Given the aforementioned effects of low-grade inflammation, ER stress, and oxidative stress, an association between obesity and organ injury (e.g., the lungs) can be inferred [65,66]. This inference is verified by the findings of the present study, which further demonstrated that the severity of lung injury (histological characteristics, injury score, and PMN infiltration) and the levels of cytokines (e.g., TNF-α and IL-1β) and lipid peroxidation in the lung tissue samples of the DIOExo group were significantly lower than those in the samples of the DIO group. These findings demonstrated the positive role of exosomes from human pcMSCs in counteracting the adverse effects of obesity. However, the findings of the present study also demonstrated that the levels of ER stress of the DIOExo and the DIO groups were not significantly different. Moreover, in the present study, the activation level of NF-κB of the DIOExo group was higher than that of the DIO group, which was an unanticipated finding. The mechanisms underlying these controversial findings require further clarification.

Our study has several limitations. Firstly, the question of whether exosomes from human pcMSCs produce similar preventive effects against sepsis in non-obese mice remains unstudied. Secondly, the question of whether exosomes from human pcMSCs can produce similar preventive effects against sepsis caused by different models of sepsis (e.g., polymicrobial sepsis induced by cecal ligation and puncture) [71,72] also remains unstudied. Thirdly, whether exosomes from human pcMSCs with multiple boluses will have long-term effects is yet to be clarified. Fourthly, the specific components (e.g., proteins, peptides, lipids, cytokines, mRNAs, and/or miRNAs) [23,24] in exosomes from human pcMSCs that contribute to the prevention of sepsis-induced lung injury in obesity have yet to be identified. Further studies need to be performed before further conclusions can be drawn.

## 5. Conclusions

Exosomes from human pcMSCs improve survivorship and mitigate acute lung injury in lipopolysaccharide-stimulated diet-induced obese mice, and the related mechanisms may involve the inhibition of ER stress, inflammation, oxidation, and apoptosis.

## Data Availability

Data is contained within the article and supplementary material.

## Supplementary Materials

The following supporting information can be downloaded at: https://www.mdpi.com/article/10.3390/antiox11040615/s1, Figure S1: Effects of high-fat diet on inducing obesity in mice; Figure S2: original images of immunoblotting assay.
