# Supplementary material for: Exosomes from Human Placenta Choriodecidual Membrane-Derived Mesenchymal Stem Cells Mitigate Endoplasmic Reticulum Stress, Inflammation, and Lung Injury in Lipopolysaccharide-Treated Obese Mice
Source: Antioxidants (Basel). 2022 Mar 23;11(4):615. doi: 10.3390/antiox11040615 (PMC9029526; doi:10.3390/antiox11040615)
Supplement: Supplementary file 1 [file antioxidants-11-00615-s001.zip › antioxidants-1626286-supplementary.pdf]

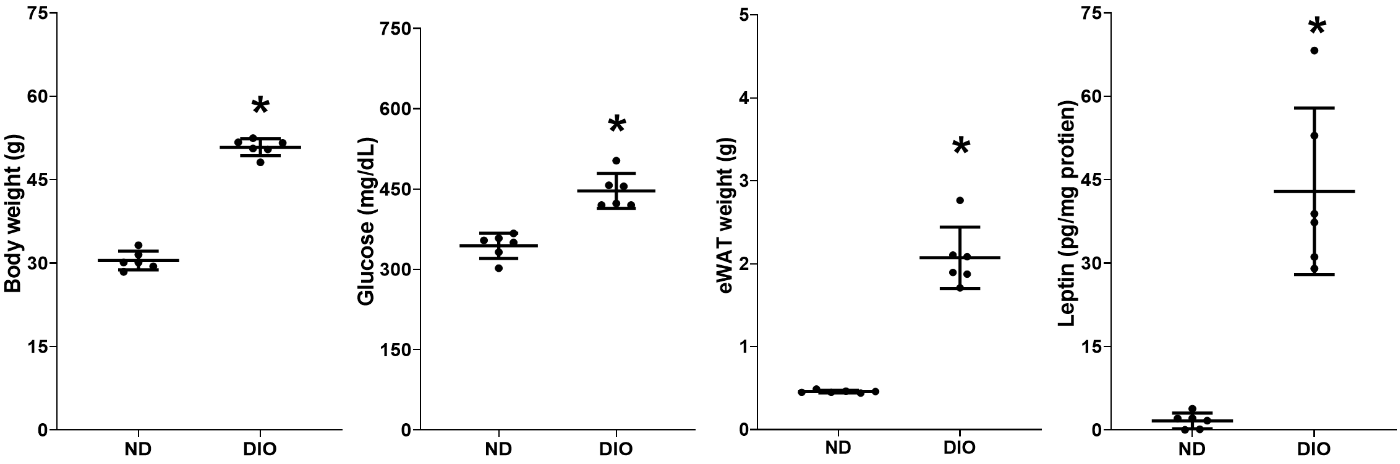

Figure S1. Body weight, blood glucose level, fat adipose weight (epididymal white adipose tissue [eWat]), and leptin concentration in high-fat diet-induced obese mice compared to normal diet non-obese mice. All assays were performed after a 12-week feeding of high-fat diet or normal diet. The leptin concentration in lung tissue was analyzed using enzyme-linked immunosorbent assay. Data were presented as means ± standard deviations. ND: the normal diet non-obese group. DIO: the high-fat diet-induced obese group. * $p < 0.05$ the DIO group versus the ND group.

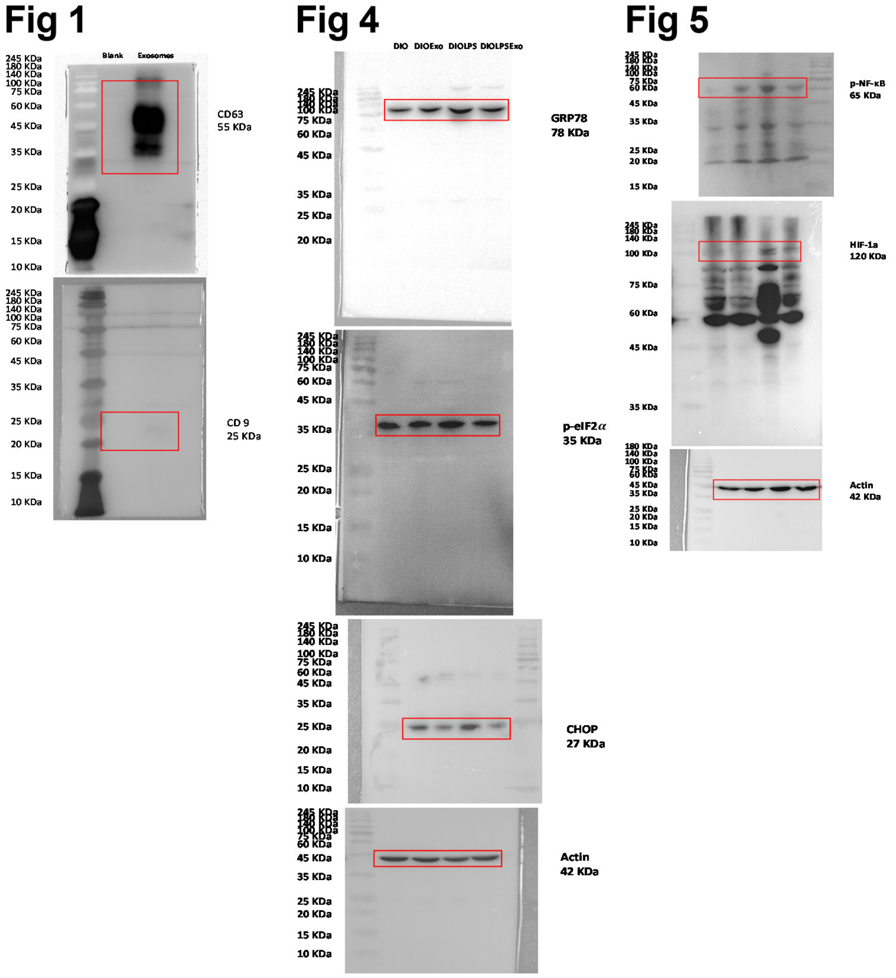

Figure S2. Uncropped representative gel photography of immunoblotting assay, presented in Figure 1, Figure 4, and Figure 5. GRP78: glucose-regulated protein 78. p-eIF2α: phosphorylated eukaryotic initiation factor 2α. CHOP: C/EBP homologous protein. p-NF-kB: phosphorylated nuclear factor-kB. HIF-1α: hypoxia-inducible factor-1α.
